# The Potential Role of Adjuvant Chemoradiotherapy in Patients with Microscopically Positive (R1) Surgical Margins after Resection of Cholangiocarcinoma

**DOI:** 10.3390/curroncol30050358

**Published:** 2023-05-04

**Authors:** Andrea Palloni, Silvia Bisello, Ilaria Maggio, Maria Massucci, Andrea Galuppi, Alessandro Di Federico, Alessandro Rizzo, Angela Dalia Ricci, Giambattista Siepe, Alessio Giuseppe Morganti, Giovanni Brandi, Giorgio Frega

**Affiliations:** 1 Medical Oncology, IRCCS Azienda Ospedaliero-Universitaria di Bologna, 40138 Bologna, Italy; andrea.palloni@aosp.bo.it (A.P.);; 2 Radiation Oncology Center, Department of Experimental, Diagnostic and Specialty Medicine—DIMES, University of Bologna, S. Orsola-Malpighi Hospital, via Giuseppe Massarenti 9, 40138 Bologna, Italy; 3 Department of Medical Oncology, Azienda USL, 40139 Bologna, Italy; 4 Department of Experimental, Diagnostic and Speciality Medicine, Sant’Orsola-Malpighi Hospital, University of Bologna, via Giuseppe Massarenti 9, 40138 Bologna, Italy; 5 Struttura Semplice Dipartimentale di Oncologia Medica per la Presa in Carico Globale del Paziente Oncologico “Don Tonino Bello”, I.R.C.C.S. Istituto Tumori “Giovanni Paolo II”, Viale Orazio Flacco 65, 70124 Bari, Italy; 6 Medical Oncology Unit, National Institute of Gastroenterology, “Saverio de Bellis” Research Hospital, 70013 Castellana Grotte, Italy; 7 Osteoncology, Soft Tissue and Bone Sarcomas, Innovative Therapy Unit, IRCCS Istituto Ortopedico Rizzoli, 40136 Bologna, Italy; giorgio.frega@ior.it

**Keywords:** cholangiocarcinoma, biliary tract cancers, chemo-radiotherapy, radiotherapy, R1 surgery

## Abstract

(1) Background: Biliary tract cancers (BTCs) are a heterogeneous group of neoplasms with dismal prognosis and the role of adjuvant chemoradiotherapy in high-risk resected patients is unclear. (2) Methods: We retrospectively analyzed the outcomes of BTC patients who received curative intent surgery with microscopically positive resection margins (R1) and adjuvant chemoradioradiotherapy (CCRT) or chemotherapy (CHT) from January 2001 to December 201. (3) Results: Out of 65 patients who underwent R1 resection, 26 received adjuvant CHT and 39 adjuvant CCRT. The median recurrence-free survival (RFS) in the CHT and CHRT groups was 13.2 and 26.8 months, respectively (*p* = 0.41). Median overall survival (OS) was higher in the CHRT group (41.9 months) as compared to the CHT group (32.2 months), but the difference was not statistically significant (HR 0.88; *p =* 0.7). A promising trend in favor of CHRT was observed in N0 patients. Finally, no statistically significant differences were observed between patients undergoing adjuvant CHRT after R1 resection and patients treated with chemotherapy alone after R0 surgery. (4) Conclusions: Our study did not show a significant survival benefit with adjuvant CHRT over CHT alone in BTC patients with positive resection margins, while a promising trend was observed.

## 1. Introduction

Biliary tract cancers (BTCs) are a heterogeneous group of rare neoplasms arising along the biliary tree and accounting for 15% to 20% of all primary hepatobiliary tumors [1]. Anatomically, BTCs are classified into intrahepatic cholangiocarcinoma (iCCA); perihilar cholangiocarcinoma (pCCA), also known as Klatskin tumor; distal extrahepatic cholangiocarcinoma (dCCA); and gallbladder cancer (GBC). Radical surgery is the only curative treatment, but only a minority of patients (20–30%) are diagnosed with resectable disease and long-term prognosis is poor [2]. Recent improvements in systemic therapy provided by the addition of immunotherapy to chemotherapy and by targeted therapy in subgroups of patients with specific molecular alteration have increased overall survival in patients with advanced disease.

In patients who undergo surgery, even after surgery with curative intent, the recurrence rate remains high, especially within the first two years after resection, and the most common pattern of recurrence involves liver and locoregional sites [3,4,5].

Microscopically positive resection margins (R1) and the presence of lymph node involvement have been identified as the main adverse prognostic factors after surgery [3,6,7,8]. In order to reduce local and systemic risk of relapse and consequently improve the long-term survival of resected patients, adjuvant strategies have been explored over the years. Recently, the randomized BILCAP trial, in adjusted ITT and per-protocol analyses, showed a significant survival benefit from six months of adjuvant capecitabine [9]; thus, it is now considered as a standard option of treatment in patients with resected biliary tract cancer [10]. In this context, the use of radiotherapy, alone or in association with chemotherapy, remains controversial. As shown by a large meta-analysis, patients with R1 resection and node-positive disease derive the greatest benefit from adjuvant chemo- or chemoradiotherapy [11]. The aim of the present study was to investigate the putative role of adjuvant concurrent chemoradiotherapy in patients with microscopically positive resection margins after surgery with curative intent.

## 2. Materials and Methods

**Figure 1:**
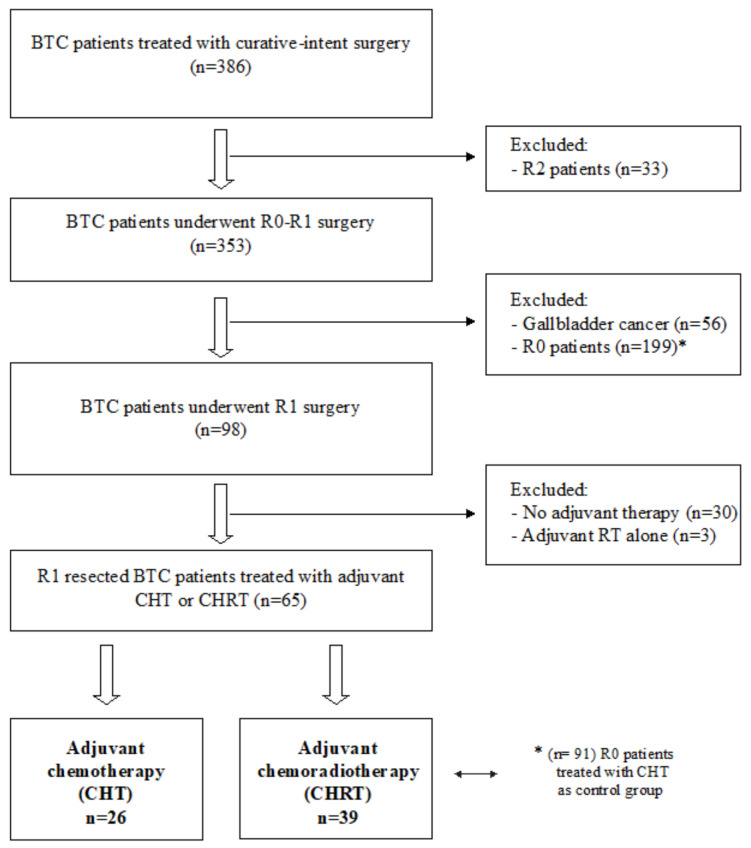
Flowchart of the study.

A total of 386 BTC patients underwent surgical resection with curative intent at S. Orsola-Malpighi University Hospital of Bologna, Italy, from January 2001 to December 2017. We excluded 33 patients with macroscopic residual disease after surgical treatment (R2), patients who did not receive any adjuvant treatment, patients who received <3 cycles of adjuvant chemotherapy or patients who received radiotherapy alone. Patients with gallbladder cancer (GBC) were also excluded, as shown in Figure 1.

We retrospectively analyzed the survival outcome and pattern of recurrence of patients with histologically proven cholangiocarcinoma who underwent R1 surgical treatment, defined by the presence of microscopically positive resection margins, followed by adjuvant chemotherapy or chemoradiotherapy. Furthermore, we included patients treated with adjuvant chemotherapy after R0 resection (*n* = 91) as a control group to be compared with the cohort of patients who underwent R1 surgery. Demographic and clinicopathologic data including age, sex and tumor-specific characteristics (site of primary tumor, histological grade and lymph node status) were collected. The pathologic stage of BTC was evaluated according to the eighth edition of the American Joint Committee on Cancer (AJCC) staging system [12]. The type of surgery varied according to tumor location. In detail, patients with intrahepatic cholangiocarcinoma (ICC) underwent anatomic liver resection, patients with perihilar CC (pCC) received bile duct resection with partial hepatectomy including the caudate lobe, and patients with distal CC underwent pancreatoduodenectomy. Regional lymphadenectomy was usually performed. We retrospectively allocated patients into two groups: adjuvant chemoradiotherapy (CHRT group) vs. chemotherapy alone (CHT group). The treatment was chosen by the attending physician, taking into account different factors such as the presence of local surgical complications, the length of post-operative recovery and patient preference. Particularly, in the case of local surgical complications such as biliary fistula or the leakage of bilioenteric anastomosis, or a postoperative performance status not permissive for concomitant treatment, radiotherapy was not administered. Adjuvant chemotherapy alone consisted of a pre-planned schedule of treatment with gemcitabine (30 min intravenous infusion at 1000 mg/m^2^ on days 1, 8, and 15 of a 28-day cycle) for a maximum of 6 cycles (6 months).

Conversely, patients in the chemoradiotherapy group were planned to receive one month of radiotherapy in 28 fractions, with the concomitant administration of low-dose gemcitabine (50 mg/m^2^ on days 1 and 5, weekly) and another five months of chemotherapy alone (with the same modalities of the other group) for a total of 6 months. Both treatments started between 4 and 8 weeks after surgery. The target volume of radiation therapy was defined according to site of the primary tumor and based on pre- and post-operative CT scan. The radiation field included regional lymph nodes (hilar, retropancreaticoduodenal, celiac and portal vein nodes) and the tumor bed. After the completion of adjuvant therapy, patients underwent a 5-year follow-up program consisting of physical examination, laboratory tests (complete blood count, serum chemistry, carcinoembryonic antigen and carbohydrate antigen 19-9) and imaging evaluation (contrast-enhanced computed tomography scan of chest, abdomen and pelvis or chest x-ray plus abdominal ultrasonography), every 4 months for the first 2 years and then every 6 months. Recurrences were classified as systemic or locoregional, defined as a relapse in the site of primary tumor and/or in the regional lymph node areas. The specific site of systemic recurrence was also recorded. Recurrence-free survival (RFS) was measured from date of surgery until recurrence and censored at tumor-related death or last follow-up visit, and overall survival (OS) as the time from surgery to tumor-related death or last follow-up visit. The study protocol was in accordance with the Declaration of Helsinki and Good Clinical Practice and received the approval of the local ethics committee with reference number CE 228/2017/O/Oss. The RFS and OS were calculated using the Cox proportional hazard model and compared using the log-rank test. Survival curves were generated using the Kaplan–Meier method. The results are presented as hazard ratios (HR) and 95% confidence intervals (CI). The chi-square test and Fisher’s exact test were used to compare the baseline characteristics among patients grouped by categorical variables. The level of critical significance was assigned at a *p*-value < 0.05. Statistical data were analyzed with R software version 1.2.5042.

## 3. Results

A total of 65 patients with BTC undergoing R1 resection were included in our retrospective analysis. Baseline patient characteristics, which were balanced between two groups, are summarized in Table 1 and Table 2.

The primary tumor location was intrahepatic in 31 patients (47.7%), perihilar in 21 (32.3%) and the distal bile duct in 13 patients (20.0%). There were 33 men (50.8%) and 32 women (49.2%) and the median age was 65.8 years (range 39.7–81.1 years). According to the eighth edition of the AJCC staging system, we observed 8 pT1 stage tumors (12.3%), 41 pT2 (63.1%), 11 pT3 (16.9%), and 5 pT4 (7.7%). Data about the tumor differentiation grade of 52 patients were available: the histological grade was G1 (well-differentiated) in two patients (3.8%) and G2 (intermediate grade) in 31 patients (59.6%), while 19 patients (36.6%) had poorly differentiated tumors (G3). Lymphadenectomy was performed in 50 patients (76.9%) and lymph node involvement was detected in 50% of them.

Median postoperative CEA values were 1.7 ng/mL in all 65 resected patients (range 0.2–6.6), 2.2 ng/mL (range 0.3–6.1) in the CHT group and 1.6 ng/mL (range 0.2–6.6) in the CHRT group.

Median postoperative CA 19.9 was 17 U/mL in the entire cohort (range 4.0–219.0). In the two adjuvant groups (CHRT and CHT), the median postoperative CA 19.9 was 16.8 U/mL (range 4.0–62.1) and 17.0 U/mL (range 5.4–219.0), respectively.

In total, 26 of 65 patients (40.0%) received adjuvant CHT with gemcitabine. The remaining 39 patients (60.0%) underwent adjuvant gem-based chemoradiotherapy (CHRT group). In the latter group, all patients received external beam radiotherapy (EBRT) while two of them also underwent brachytherapy. The median radiation dose was 5040 cGy. After a median follow-up period of 35.2 months, disease recurrence occurred in 46 patients (70.8%). The site of disease relapse was locoregional in 26 patients.

No statistically significant difference in the recurrence patterns between the two groups was observed (incidence of local recurrence was 14 in the CHT group vs. 12 in the CHRT group).

The first recurrence pattern of patients who received adjuvant CHRT was intrahepatic in 16 cases (57.5%), locoregional extrahepatic in 8 patients (28.5%), peritoneal carcinomatosis in 5 (18.5%) and lung metastases in 6 patients (21.5%). For R1 patients treated with adjuvant CHT, the first sites of relapse were locoregional extrahepatic in 15 patients (71.4%), intrahepatic in 4 (20.0%), lung in 4 patients (20.0%), peritoneum in 2 (9.5%) and bone in 1 patient (4.7%). In total, 6 out of 39 patients (15.4%) in the CHRT group and 7 out of 20 patients in the CHT group (35.0%) experienced first recurrence in multiple sites.

The median RFS and OS of the entire cohort were 13.9 and 39.3 months, respectively.

**Figure 2:**
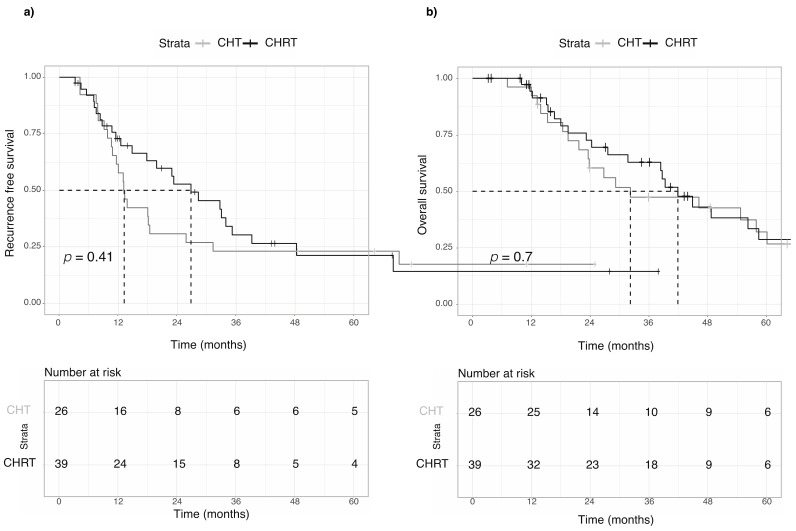
Kaplan–Meier curves comparing different adjuvant strategies after upfront surgery. (**a**) Recurrence-free survival of patients treated with chemotherapy alone or combined chemoradiotherapy. (**b**) Overall survival of patients treated with chemotherapy alone or combined chemoradiotherapy.

Analyzing the two groups of treatment, we observed a median RFS of 13.2 months and 26.8 months in the CHT group and CHRT group, respectively (HR 0.78; 95% CI 0.43–1.4, *p* = 0.41). One-year RFS was 58% in the CHT group and 73% in the CHRT group, as shown in Figure 2a.

Patients treated with chemotherapy alone had a median OS of 32.2 months, as compared to 41.9 months of median OS in the CHRT group (HR 0.88; 95% CI 0.46–1.66, *p =* 0.7). Two-year OS was 60.3% in the CHT group and 72.6% in the CHRT group, as shown in Figure 2b.

Given the small number of patients in our series, the mild imbalance in patients’ characteristics between the CHT and CHRT groups could have a significant impact on the outcome, even if the difference was not statistically significant. We did not observe a statistically significant difference in the toxicity profile between the two groups. We performed a multivariate analysis including survival variables such as the type of adjuvant regimen (CHRT vs. CHT), site of primary tumor, lymph node status, T stage and tumor grade. The only independent prognostic factor significantly associated with recurrence-free survival was the presence of lymph node involvement (HR 3.06; 95% CI 1.38–6.8, *p* = 0.006) (Supplementary Table S1).

**Figure 3:**
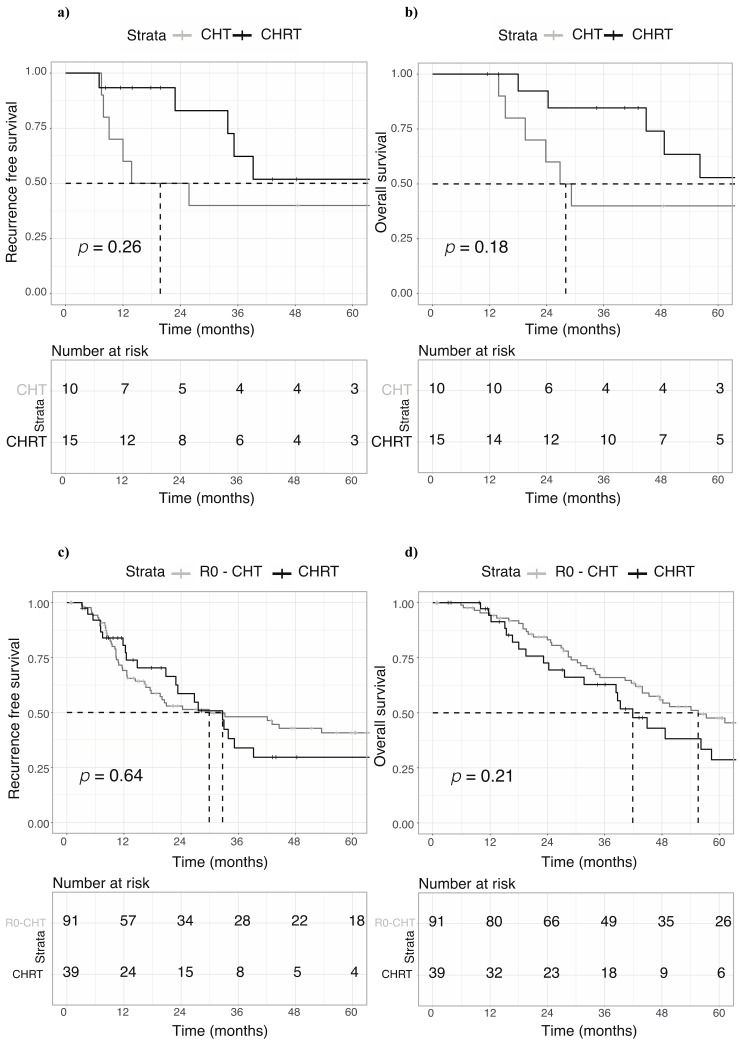
Kaplan–Meier curves comparing different adjuvant strategies after upfront surgery in patients without nodal involvement (N0). (**a**) Recurrence-free survival of N0 patients treated with chemotherapy alone or combined chemoradiotherapy. (**b**) Overall survival of N0 patients treated with chemotherapy alone or combined chemoradiotherapy. (**c**) Recurrence-free survival of patients treated with combined chemoradiotherapy after R1 resection compared with an independent retrospective cohort of patients (*n* = 91) who received only CTX after R0 surgery. (**d**) Overall survival of patients treated with combined chemoradiotherapy after R1 resection compared with an independent retrospective cohort of patients (*n* = 91) who received only CTX after R0 surgery.

We further focused on N0 patients and still observed no significant results, but with a promising trend in RFS (HR 0.54; 95% CI 0.18–1.61, *p* = 0.26) and OS (HR 0.48; 95% CI 0.16–1.43, *p* = 0.18), in favor of the chemoradiotherapy group (Figure 3a,b).

Analyzing these data, we must bear in mind the small number of patients included. Finally, we compared the outcome of 39 patients treated with CHRT with an independent retrospective cohort of R0 patients (*n* = 91) who received only CHT. No statistically significant differences were observed between the two groups in terms of RFS (mRFS 29.9 vs. 32.7 months, respectively; HR 1.13; 95% CI 0.67–1.9, *p* = 0.64) and OS (mOS 41.9 vs. 55.6 months, respectively; HR 1.39; 95% CI 0.83–2.33, *p* = 0.21) (Figure 3c,d).

## 4. Discussion

Surgical resection is the only potentially curative treatment for biliary tract cancer but, even after radical surgery, recurrence occurs in more than half of patients [13] and negatively impacts long-term outcome. Together with the presence of lymph node involvement, the surgical margin status is the main prognostic factor in resected patients [6,7,8,14,15,16,17]. Prior to 2017, adjuvant therapy in BTC was supported by a large meta-analysis showing a significant benefit in patients with lymph node involvement (OR, 0.49; *p* = 0.004) and microscopic positive resection margins (R1) (OR, 0.36; *p* = 0.002) [11]. Since 2017, the year that the BILCAP trial results were presented, capecitabine has been considered as the standard of care in this setting [9]. BILCAP was a large randomized phase III trial, conducted in the United Kingdom, which randomly allocated 447 BTC patients to adjuvant capecitabine or observation. The overall population consisted mainly of eCCAs, representing about 60% of all subjects in both arms; the proportions of R1 and N1 patients were 38% and 46%, respectively. A pre-specified ITT sensitivity analysis was adjusted for grading, nodal involvement, and gender (HR 0.71, 95% CI 0.55–0.92; *p* = 0.010) and the per-protocol population survival analysis showed a significant benefit with adjuvant capecitabine over observation, obtaining an improvement in median OS (from 36 to 53 months, HR 0.75; *p* = 0.028) and RFS (from 17.5 to 24.4 months, HR 0.75; *p* = 0.033) [9].

Based on the results of a phase III multicentric randomized Japanese trial (JCOG1202, ASCOT), another oral fluoropyrimidine derivative, S-1, was associated with significantly improved overall survival compared to observation in patients with resected BTC [18]. However, while it could represent the standard of care in an adjuvant setting for Asian BTC patients, the same conclusions cannot be applied to Western countries.

Conversely, despite its consolidated role in metastatic disease, gemcitabine does not represent a valid therapeutic choice as adjuvant treatment, since recent randomized trials showed negative results [19,20].

The BCAT trial included only patients with resected extrahepatic biliary tract cancer and randomized them to receive adjuvant gemcitabine or observation alone after surgery. The proportion of patients with a positive lymph node status was 35.9% in the gemcitabine arm and 33.3% in the observation arm, similar to our series (38.5% of N1 patients in the entire cohort). At the same time, the rate of R1-resection ranged from 9.4% in the gem arm to 13.0% in the observation arm, and this low percentage may suggest a population of resected patients with good prognosis. The survival analysis did not show any significant differences in OS (median OS 62.3 months of gemcitabine vs. 63.8 months of observation, *p* = 0.964) and in RFS (median RFS 36.0 vs. 39.9 months, respectively, *p* = 0.693) [19].

Gemcitabine also failed to demonstrate a role as adjuvant treatment in association with oxaliplatin. The PRODIGE-12/ACCORD-18 trial randomized 196 BTC patients to gemcitabine and oxaliplatin (GemOx) or observation alone after macroscopically complete resection (R0/R1). Differently from the BCAT trial, PRODIGE-12/ACCORD-18 enrolled all types of BTC, including iCCA, eCCA and GBC, with a predominance of intrahepatic cholangiocarcinomas (44% of all included subjects). After a median follow-up of 46.5 months, no statistically significant differences were observed between the two arms in terms of RFS (HR 0.83; *p* = 0.31) and OS (HR 1.08; *p* = 0.74) [20]. Lamarca et al. noted that one of the supposed explanations of the negative results of this trial compared to the positive findings of BILCAP could be the higher proportion of patients with R1 and N1 disease, the most important prognostic factors in resected BTC, in the latter study [21].

Future evidence of adjuvant combination chemotherapy may be derived by the ongoing ACTICCA-1 randomized phase III clinical trial comparing the association of cisplatin and gemcitabine to capecitabine as adjuvant treatment strategy in patients with resected biliary tract cancer, including intrahepatic, hilar or extrahepatic cholangiocarcinoma or muscle-invasive gallbladder carcinoma (NCT02170090).

Even if recent results from large randomized trials show that immunotherapy significantly increases survival as compared to chemotherapy alone in advanced biliary tract cancer, its role in the adjuvant setting has not yet been proven [22,23].

The rationale of adding radiotherapy to adjuvant chemotherapy is to reduce the risk of locoregional recurrence, which represents the predominant pattern of relapse in patients with microscopically involved resection margins and the leading cause of morbidity and tumor-related mortality [24]. Even in the most experienced hepatobiliary centers, the incidence of microscopically positive resection margins (R1) in the historical BTC series remains common, given the complex surgical anatomy and the local tumor growth pattern in the biliary tree.

To date, there are no large prospective randomized trials addressing the potential benefit of adjuvant radiotherapy in patients with resected biliary tract cancer.

Retrospective studies reported improvements in mOS and 5-year OS (from 8 to 24 months and from 13.5% to 33.9%, respectively) in patients who received radiotherapy alone after curative-intent surgery, even with the limitations of the low number of cases and the heterogeneity of treatment [25,26,27,28]. The retrospective study by Cheng et al. investigated the survival of 75 consecutive HCCA patients undergoing curative-intent surgery for Klatskin tumors (pCCA). After multivariate analysis, a highly significant survival benefit was observed in favor of adjuvant RT, especially in patients who received R1/R2 resection [29].

A Surveillance, Epidemiology and End Results analysis of 3839 patients with IHCC showed a median OS of 11 months with surgery followed by adjuvant radiotherapy versus 6 months with surgery alone (*p* = 0.014) [30].

The meta-analysis by Horgan et al., including 20 studies and a total of 6712 patients, showed that patients who received adjuvant chemoradiotherapy had a greater survival benefit as compared to those treated with radiotherapy alone (OR, 0.61; 95% CI, 0.38 to 0.99; *p* = 0.049) [11].

Focusing on the chemoradiotherapy combination, scientific evidence is limited and inconclusive [31,32], with the majority of data provided by retrospective studies and population-based registry analyses characterized by the heterogeneity of treatments and patients’ selection bias.

For example, in one of these studies, 43% of the patients who composed the radiotherapy group received only palliative surgery and, when a complete resection was obtained, a higher rate of portal vein or hepatic artery involvement occurred [31].

Encouraging data are predominantly reported in extrahepatic and gallbladder tumors. In patients with eCCA, a meta-analysis by Bonet Beltràn et al., including studies with radiotherapy alone or in combination with chemotherapy, showed a significant survival benefit for adjuvant radiotherapy compared to surgery alone (HR 0.62; 95%CI 0.48–0.78, *p* < 0.001), especially in patients with positive margins [33].

Recently, a retrospective study investigated the outcome of 92 BTC patients, about half of whom were affected by distal bile duct cancer, who underwent adjuvant chemoradiotherapy or chemotherapy after curative-intent surgery at Keimyung University Dongsan Medical Center. In contrast with our series, chemotherapeutic regimens used as monotherapy or as a concurrent drug during radiotherapy were heterogeneous, and included 5-fluorouracil (5-FU), 5-FU/cisplatin, gemcitabine and gemcitabine/cisplatin. In the overall population, the CHRT group had a significantly longer RFS than the CHT group, while a non-significant mild OS benefit was observed. In the subgroup of patients with positive resection margins, no statistically significant OS and RFS differences were observed in favor of CHRT over CHT [34].

Prospective evidence for CHRT in eCCA and gallbladder cancer came from the phase II SWOG S0809 study, where patients with resected pT2–4, N1 tumors or positive surgical margins received gemcitabine and capecitabine followed by RT with concurrent capecitabine. The survival rates were significantly higher than those reported in historical controls and treatment was well tolerated [35].

In the same study, authors reported that 9 out of 14 patients (18%) who developed disease relapse during follow-up experienced locoregional recurrence, and 9 of them had concurrent distant relapse [30]. In a secondary analysis of the SWOG S0809 trial, Gholami et al. investigated the survival outcomes of 69 patients who completed adjuvant radiotherapy, according to their nodal status. No statistically significant differences were observed between patients with node-negative (N0) and node-positive disease (N+) in terms of OS and DSF, even if 2-year OS and DFS were higher in the first group. In addition, the authors observed a higher rate of distant failure (42.2% vs. 25.0%, *p* = 0.04) in N+ vs. N0 tumors, and they concluded that adjuvant chemoradiation may influence local control disease in patients with node-positive disease after surgery [36]. A retrospective study on 65 resected extrahepatic BTCs aimed to investigate whether adjuvant concurrent chemoradiation improves survival and locoregional disease control in patients with high-risk resected extrahepatic biliary tract cancer. The authors showed similar outcomes between the group of N0R0 patients who underwent surgery alone and the group of high-risk patients (N1R1) treated with adjuvant chemoradiotherapy [37].

Despite the low level of evidence summarized above, adjuvant chemoradiotherapy is a recommended option for patients who have undergone R1 resection [32].

In the overall population of our study, we did not report statistically significant differences in RFS and OS between R1 patients treated with CHRT and CHT alone, but a positive trend in the CHRT subgroup was observed. In our series, chemoradiotherapy seems to not improve RFS and OS, while a non-significant positive trend emerged in the small subgroup of node-negative patients. In addition, as shown by the multivariate analysis, the only independent prognostic factor significantly associated with recurrence-free survival in R1 patients was lymph node status. An analysis performed comparing our R1 CHRT-treated patients with a historical cohort of R0 patients receiving only chemotherapy showed non-significant differences in survival outcomes, suggesting a potential role of chemoradiotherapy in partially dampening the negative prognostic value of positive resection margins.

In gastrointestinal tumors, it is known that an adequate recovery from surgery before starting adjuvant therapy may improve tolerance of treatment [38].

On the other hand, as shown in resected BTC patients, a treatment delay beyond a median time point of 59 days after curative-intent surgery was associated with a significant decrement in overall survival. In our study, the timing of adjuvant treatment reflects this evidence, since all patients included in the analysis started adjuvant treatment within 60 days after surgery, after at least 4 weeks from resection [39].

Even if no significant difference in recurrence pattern was observed between the cohort of patients who received adjuvant CHRT and the cohort of patients who received only CHT, a higher rate of extrahepatic locoregional recurrence as the first site of relapse was observed in the CHT group compared to the CHRT group. We supposed that adding radiotherapy to adjuvant chemotherapy in patients with microscopically positive (R1) margin resection may influence local extrahepatic control, given the extent of the radiation field at regional lymph nodes and tumor bed, and this may delay the clinical relevance of local recurrence, which affects the patient’s quality of life more than distant recurrence.

The choice of the specific chemotherapeutic regimen used in association with radiotherapy remains an open issue, given the limited and heterogeneous data. While concurrent radiotherapy (RT) plus infusional FU or capecitabine is widely used for gastrointestinal tract malignancies, gemcitabine represents a valid radiosensitizing agent in the adjuvant setting for pancreatic cancer.

In light of the results of the BILCAP trial and the negative conclusions of the BCAT and PRODIGE 12 trials, we believe that new strategies of CHRT will mostly focus on the combination of RT with 5-FU (or its precursor capecitabine) rather than gemcitabine. For this reason, the presence of gemcitabine as a chemotherapeutic agent in our series, even if justified by the treatment of patients in a pre-BILCAP period, represents a significant limitation of our analysis.

In addition, given the prevalence of iCCA in our study, we could not apply the same conclusions on the role of chemoradiotherapy to a subgroup of R1-resected extrahepatic tumors, which is the most represented CC population in different studies about adjuvant radiotherapy.

Nevertheless, and even with the limitation of a small and retrospective series, our data seem to anyhow support prospective investigations through randomized controlled trials (RCT) to validate the potential role of RT in addition to adjuvant chemotherapy in the specific high-risk group of resected BTC patients with a positive surgical margin.

## Figures and Tables

**Table 1:** Baseline characteristics of patients who underwent R1 resection.

	Total (*n* = 65)	Adjuvant CHT (*n* = 26)	Adjuvant CHRT (*n* = 39)	*p* Value
Age, years				
≥70 (%)	21 (32.2)	10 (38.4)	11 (28.2)	0.39
<70 (%)	44 (67.7)	16 (61.6)	28 (71.8)	
Median	65.8	67	64.4	
range	39.7–81.8	39.7–81.8	43.2–80.8	
Sex (%)				
M	33 (50.8)	12 (46.1)	21 (53.8)	0.54
F	32 (49.2)	14 (53.9)	18 (46.2)	
Site of primary tumor				
ICC	31 (47.7)	15 (57.7)	16 (41.0)	0.41
pCC	21 (32.3)	7 (26.9)	14 (35.9)	
dCC	13 (20.0)	4 (15.4)	9 (23.1)	
Grading (%)				
G1	2 (3.1)	2 (7.8)	0 (0.0)	0.33
G2	31 (47.7)	10 (38.4)	21 (53.8)	
G3	19 (29.2)	7 (26.9)	12 (30.8)	
n.a.	13 (20.0)	7 (26.9)	6 (15.4)	
Pathological lymph node status (%)				
N0	25 (38.5)	10 (38.4)	15 (38.5)	0.41
N+	25 (38.5)	8 (30.8)	17 (43.6)	
n.a.	15 (23.0)	8 (30.8)	7 (17.9)	
T stage (8th edition AJCC staging system) (%)				
T1	8 (12.3)	5 (19.2)	3 (7.7)	0.17
T2	41 (63.1)	18 (69.2)	23 (59.0)	
T3	11 (16.9)	2 (7.7)	9 (23.1)	
T4	5 (7.7)	1 (3.9)	4 (10.2)	

**Table 2:** CEA and CA 19.9 values of patients who underwent R1 resection.

	Total (*n* = 65)	Adjuvant CHT (*n* = 26)	Adjuvant CHRT (*n* = 39)
**Postoperative CEA**			
Median (ng/mL)	1.7	2.2	1.6
Range (ng/mL)	0.2–6.6	0.3–6.1	0.2–6.6
**Postoperative CA 19.9**			
Median (U/mL)	17	16.8	17
Range (U/mL)	4.0–219.0	4.0–62.1	5.4–219.0

Abbreviations: CA19-9, cancer antigen 19-9; CEA, carcinoembryonic antigen.

## Data Availability

The data presented in this study are available on request from the corresponding author.

## Supplementary Materials

The following supporting information can be downloaded at: https://www.mdpi.com/article/10.3390/curroncol30050358/s1, Table S1: Multivariate analysis of recurrence-free survival for R1 patients.

## Abbreviations

iCCA	intrahepatic cholangiocarcinoma
dCCA	distal cholangiocarcinoma
pCCA	perihilar cholangiocarcinoma
CHRT	chemoradiotherapy
CHT	chemotherapy
BTC	biliary tract cancer
GBC	gallbladder cancer
OS	overall survival
RFS	recurrence-free survival

## References

[1] Blechacz B. (2017). Cholangiocarcinoma: Current Knowledge and New Developments. Gut Liver.

[2] Doherty B., Nambudiri V.E., Palmer W.C. (2017). Update on the Diagnosis and Treatment of Cholangiocarcinoma. Curr. Gastroenterol. Rep..

[3] Jarnagin W.R., Ruo L., Little S.A., Klimstra D., D’Angelica M., DeMatteo R.P., Wagman R., Blumgart L.H., Fong Y. (2003). Patterns of Initial Disease Recurrence after Resection of Gallbladder Carcinoma and Hilar Cholangiocarcinoma: Implications for Adjuvant Therapeutic Strategies. Cancer.

[4] Groot Koerkamp B., Wiggers J.K., Allen P.J., Besselink M.G., Blumgart L.H., Busch O.R.C., Coelen R.J., D’Angelica M.I., DeMatteo R.P., Gouma D.J. (2015). Recurrence Rate and Pattern of Perihilar Cholangiocarcinoma after Curative Intent Resection. J. Am. Coll. Surg..

[5] Spolverato G., Kim Y., Alexandrescu S., Marques H.P., Lamelas J., Aldrighetti L., Gamblin T.C., Maithel S.K., Pulitano C., Bauer T.W. (2016). Management and Outcomes of Patients with Recurrent Intrahepatic Cholangiocarcinoma Following Previous Curative-Intent Surgical Resection. Ann. Surg. Oncol..

[6] Farges O., Fuks D., Boleslawski E., Le Treut Y.-P., Castaing D., Laurent A., Ducerf C., Rivoire M., Bachellier P., Chiche L. (2011). Influence of Surgical Margins on Outcome in Patients with Intrahepatic Cholangiocarcinoma: A Multicenter Study by the AFC-IHCC-2009 Study Group. Ann. Surg..

[7] DeOliveira M.L., Cunningham S.C., Cameron J.L., Kamangar F., Winter J.M., Lillemoe K.D., Choti M.A., Yeo C.J., Schulick R.D. (2007). Cholangiocarcinoma: Thirty-One-Year Experience with 564 Patients at a Single Institution. Ann. Surg..

[8] De Jong M.C., Nathan H., Sotiropoulos G.C., Paul A., Alexandrescu S., Marques H., Pulitano C., Barroso E., Clary B.M., Aldrighetti L. (2011). Intrahepatic Cholangiocarcinoma: An International Multi-Institutional Analysis of Prognostic Factors and Lymph Node Assessment. J. Clin. Oncol..

[9] Primrose J.N., Fox R.P., Palmer D.H., Malik H.Z., Prasad R., Mirza D., Anthony A., Corrie P., Falk S., Finch-Jones M. (2019). Capecitabine Compared with Observation in Resected Biliary Tract Cancer (BILCAP): A Randomised, Controlled, Multicentre, Phase 3 Study. Lancet Oncol..

[10] Malka D., Edeline J. (2019). Adjuvant Capecitabine in Biliary Tract Cancer: A Standard Option?. Lancet Oncol..

[11] Horgan A.M., Amir E., Walter T., Knox J.J. (2012). Adjuvant Therapy in the Treatment of Biliary Tract Cancer: A Systematic Review and Meta-Analysis. J. Clin. Oncol..

[12] Edge S.B., American Joint Committee on Cancer (2010). AJCC Cancer Staging Manual.

[13] Jung S.J., Woo S.M., Park H.K., Lee W.J., Han M.A., Han S.-S., Kim S.H., Park S.-J., Kim T.H., Koh Y.H. (2012). Patterns of Initial Disease Recurrence after Resection of Biliary Tract Cancer. Oncology.

[14] Uchiyama K., Yamamoto M., Yamaue H., Ariizumi S., Aoki T., Kokudo N., Ebata T., Nagino M., Ohtsuka M., Miyazaki M. (2011). Impact of Nodal Involvement on Surgical Outcomes of Intrahepatic Cholangiocarcinoma: A Multicenter Analysis by the Study Group for Hepatic Surgery of the Japanese Society of Hepato-Biliary-Pancreatic Surgery. J. Hepatobiliary Pancreat. Sci..

[15] Ribero D., Pinna A.D., Guglielmi A., Ponti A., Nuzzo G., Giulini S.M., Aldrighetti L., Calise F., Gerunda G.E., Tomatis M. (2012). Surgical Approach for Long-Term Survival of Patients With Intrahepatic Cholangiocarcinoma: A Multi-Institutional Analysis of 434 Patients. Arch. Surg..

[16] Jarnagin W.R., Fong Y., DeMatteo R.P., Gonen M., Burke E.C., Bodniewicz B.S.J., Youssef B.A.M., Klimstra D., Blumgart L.H. (2001). Staging, Resectability, and Outcome in 225 Patients With Hilar Cholangiocarcinoma. Ann. Surg..

[17] Ueda J., Yoshida H., Mamada Y., Taniai N., Yoshioka M., Hirakata A., Kawano Y., Mizuguchi Y., Shimizu T., Kanda T. (2018). Evaluation of Positive Ductal Margins of Biliary Tract Cancer in Intraoperative Histological Examination. Oncol. Lett..

[18] Nakachi K., Ikeda M., Konishi M., Nomura S., Katayama H., Kataoka T., Todaka A., Yanagimoto H., Morinaga S., Kobayashi S. (2023). Adjuvant S-1 Compared with Observation in Resected Biliary Tract Cancer (JCOG1202, ASCOT): A Multicentre, Open-Label, Randomised, Controlled, Phase 3 Trial. Lancet.

[19] Ebata T., Hirano S., Konishi M., Uesaka K., Tsuchiya Y., Ohtsuka M., Kaneoka Y., Yamamoto M., Ambo Y., Shimizu Y. (2018). Randomized Clinical Trial of Adjuvant Gemcitabine Chemotherapy versus Observation in Resected Bile Duct Cancer. Br. J. Surg..

[20] Edeline J., Benabdelghani M., Bertaut A., Watelet J., Hammel P., Joly J.-P., Boudjema K., Fartoux L., Bouhier-Leporrier K., Jouve J.-L. (2019). Gemcitabine and Oxaliplatin Chemotherapy or Surveillance in Resected Biliary Tract Cancer (PRODIGE 12-ACCORD 18-UNICANCER GI): A Randomized Phase III Study. J. Clin. Oncol..

[21] Lamarca A., Edeline J., McNamara M.G., Hubner R.A., Nagino M., Bridgewater J., Primrose J., Valle J.W. (2020). Current Standards and Future Perspectives in Adjuvant Treatment for Biliary Tract Cancers. Cancer Treat. Rev..

[22] Kelley R.K., Ueno M., Yoo C., Finn R.S., Furuse J., Ren Z., Yau T., Klümpen H.-J., Chan S.L., Ozaka M. (2023). Pembrolizumab in Combination with Gemcitabine and Cisplatin Compared with Gemcitabine and Cisplatin Alone for Patients with Advanced Biliary Tract Cancer (KEYNOTE-966): A Randomised, Double-Blind, Placebo-Controlled, Phase 3 Trial. Lancet.

[23] Oh D.-Y., Ruth He A., Qin S., Chen L.-T., Okusaka T., Vogel A., Kim J.W., Suksombooncharoen T., Ah Lee M., Kitano M. (2022). Durvalumab plus Gemcitabine and Cisplatin in Advanced Biliary Tract Cancer. NEJM Evid..

[24] Komaya K., Ebata T., Yokoyama Y., Igami T., Sugawara G., Mizuno T., Yamaguchi J., Nagino M. (2018). Recurrence after Curative-Intent Resection of Perihilar Cholangiocarcinoma: Analysis of a Large Cohort with a Close Postoperative Follow-up Approach. Surgery.

[25] Pitt H.A., Nakeeb A., Abrams R.A., Coleman J., Piantadosi S., Yeo C.J., Lillemore K.D., Cameron J.L. (1995). Perihilar Cholangiocarcinoma. Postoperative Radiotherapy Does Not Improve Survival. Ann. Surg..

[26] Todoroki T., Ohara K., Kawamoto T., Koike N., Yoshida S., Kashiwagi H., Otsuka M., Fukao K. (2000). Benefits of Adjuvant Radiotherapy after Radical Resection of Locally Advanced Main Hepatic Duct Carcinoma. Int. J. Radiat. Oncol. Biol. Phys..

[27] Gwak H.K., Kim W.C., Kim H.J., Park J.H. (2010). Extrahepatic Bile Duct Cancers: Surgery Alone versus Surgery plus Postoperative Radiation Therapy. Int. J. Radiat. Oncol. Biol. Phys..

[28] Vern-Gross T.Z., Shivnani A.T., Chen K., Lee C.M., Tward J.D., MacDonald O.K., Crane C.H., Talamonti M.S., Munoz L.L., Small W. (2011). Survival Outcomes in Resected Extrahepatic Cholangiocarcinoma: Effect of Adjuvant Radiotherapy in a Surveillance, Epidemiology, and End Results Analysis. Int. J. Radiat. Oncol. Biol. Phys..

[29] Cheng Q., Luo X., Zhang B., Jiang X., Yi B., Wu M. (2007). Predictive Factors for Prognosis of Hilar Cholangiocarcinoma: Postresection Radiotherapy Improves Survival. Eur. J. Surg. Oncol..

[30] Shinohara E.T., Mitra N., Guo M., Metz J.M. (2008). Radiation Therapy Is Associated with Improved Survival in the Adjuvant and Definitive Treatment of Intrahepatic Cholangiocarcinoma. Int. J. Radiat. Oncol. Biol. Phys..

[31] Nakeeb A., Tran K.Q., Black M.J., Erickson B.A., Ritch P.S., Quebbeman E.J., Wilson S.D., Demeure M.J., Rilling W.S., Dua K.S. (2002). Improved Survival in Resected Biliary Malignancies. Surgery.

[32] Nelson J.W., Ghafoori A.P., Willett C.G., Tyler D.S., Pappas T.N., Clary B.M., Hurwitz H.I., Bendell J.C., Morse M.A., Clough R.W. (2009). Concurrent Chemoradiotherapy in Resected Extrahepatic Cholangiocarcinoma. Int. J. Radiat. Oncol. Biol. Phys..

[33] Bonet Beltrán M., Allal A.S., Gich I., Solé J.M., Carrió I. (2012). Is Adjuvant Radiotherapy Needed after Curative Resection of Extrahepatic Biliary Tract Cancers? A Systematic Review with a Meta-Analysis of Observational Studies. Cancer Treat. Rev..

[34] Kim H., Heo M.H., Kim J.Y. (2020). Comparison of the Effects of Adjuvant Concurrent Chemoradiotherapy and Chemotherapy for Resected Biliary Tract Cancer. BMC Gastroenterol..

[35] Ben-Josef E., Guthrie K.A., El-Khoueiry A.B., Corless C.L., Zalupski M.M., Lowy A.M., Thomas C.R., Alberts S.R., Dawson L.A., Micetich K.C. (2015). SWOG S0809: A Phase II Intergroup Trial of Adjuvant Capecitabine and Gemcitabine Followed by Radiotherapy and Concurrent Capecitabine in Extrahepatic Cholangiocarcinoma and Gallbladder Carcinoma. J. Clin. Oncol..

[36] Gholami S., Colby S., Horowitz D.P., Guthrie K.A., Ben-Josef E., El-Khoueiry A.B., Blanke C.D., Philip P.A., Kachnic L.A., Ahmad S.A. (2023). ASO Visual Abstract: Adjuvant Chemoradiation in Patients with Lymph Node-Positive Biliary Tract Cancers-Secondary Analysis of a Single-Arm Clinical Trial (SWOG 0809). Ann. Surg. Oncol..

[37] Shroff R.T., Kennedy E.B., Bachini M., Bekaii-Saab T., Crane C., Edeline J., El-Khoueiry A., Feng M., Katz M.H.G., Primrose J. (2019). Adjuvant Therapy for Resected Biliary Tract Cancer: ASCO Clinical Practice Guideline. J. Clin. Oncol..

[38] Valle J.W., Palmer D., Jackson R., Cox T., Neoptolemos J.P., Ghaneh P., Rawcliffe C.L., Bassi C., Stocken D.D., Cunningham D. (2014). Optimal Duration and Timing of Adjuvant Chemotherapy after Definitive Surgery for Ductal Adenocarcinoma of the Pancreas: Ongoing Lessons from the ESPAC-3 Study. J. Clin. Oncol..

[39] Parsons M., Lloyd S., Johnson S., Scaife C., Soares H., Kim R., Kim R., Garrido-Laguna I., Tao R. (2022). The Implications of Treatment Delays in Adjuvant Therapy for Resected Cholangiocarcinoma Patients. J. Gastrointest. Cancer.

