# Supplementary figures and images for: The Potential Role of Adjuvant Chemoradiotherapy in Patients with Microscopically Positive (R1) Surgical Margins after Resection of Cholangiocarcinoma
Source: Curr Oncol. 2023 May 4;30(5):4754–66. doi: 10.3390/curroncol30050358 (PMC10217181; doi:10.3390/curroncol30050358)

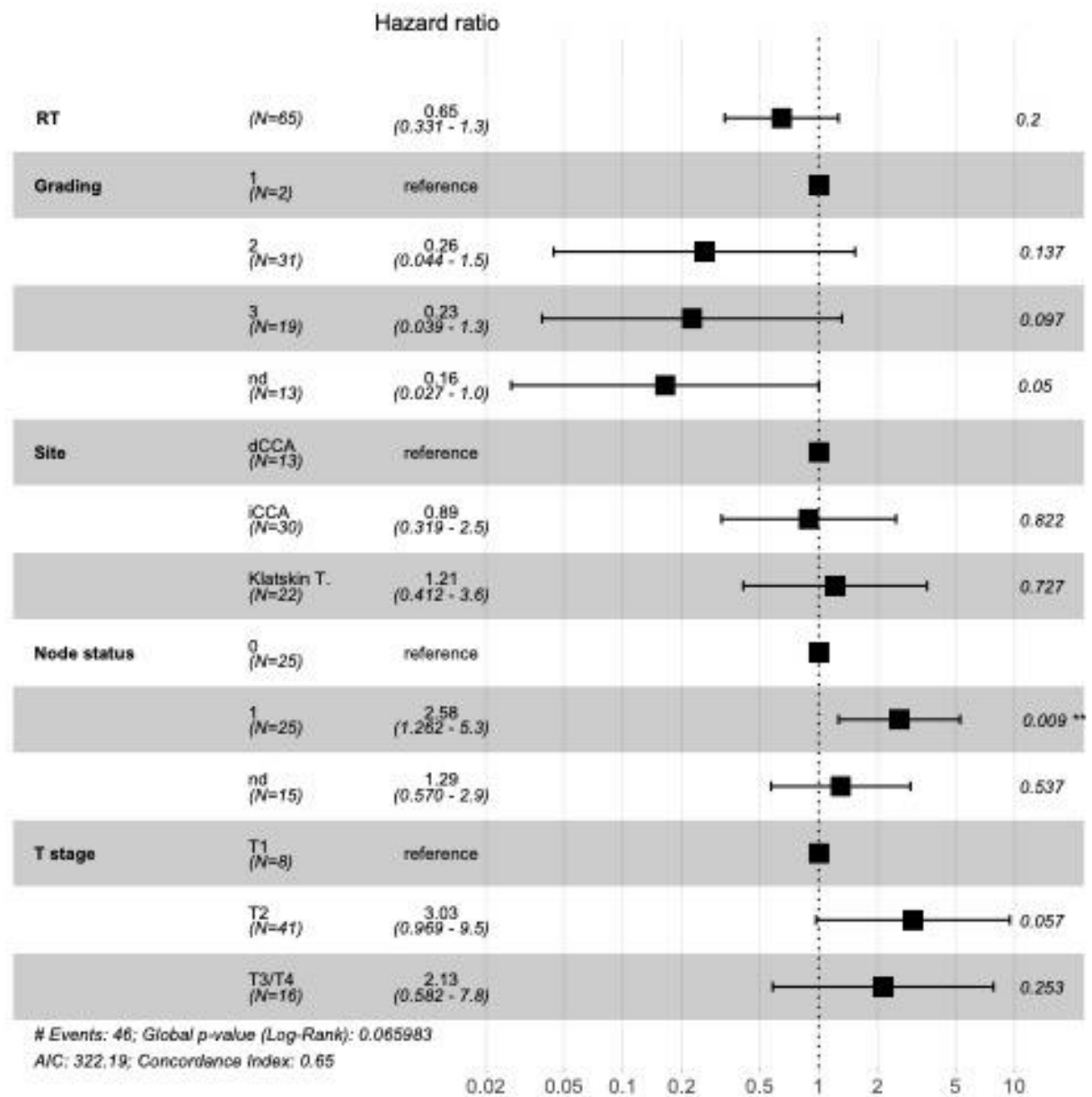

Supplementary Table S1. Multivariate analysis of recurrence-free survival for R1 patients.

Supplement: Supplementary file 1 [file curroncol-30-00358-s001.zip › curroncol-2313613-supplementary.pdf]
